# Retraction: The impact of high-intensity exercise on patients with depression: a systematic review and meta-analysis of randomized controlled trials

**DOI:** 10.3389/fpubh.2026.2026366

**Published:** 2026-10-14

**Authors:** Frontiers Editorial Office

The journal retracts the 13 August 2025 article cited above.

Following publication, concerns were raised regarding the scientific validity of the article. An investigation was conducted in accordance with Frontiers' policies.

The authors failed to provide a satisfactory explanation and as a result, the conclusions of the article have been deemed unreliable, and the article has been retracted.

This retraction was approved by the Chief Editors of Frontiers in Public Health and the Chief Executive Editor of Frontiers. The authors agree to this retraction.

Frontiers would like to thank the concerned readers who contacted us regarding the published article.

